# New York Odontological Society

**Published:** 1887-05

**Authors:** 


### NEW YORK ODONTOLOGICAL SOCIETY.

Reported for the Independent Practitioner.

The April meeting of this society was held on Tuesday evening, the 12th ult., in the parlors of the Academy of Medicine, Vice-President Dr. J. Morgan Howe occupying the chair.

The President, Dr. E. A. Bogue, read a voluminous but exceedingly interesting paper written for and sent to the society by Dr. I. B. Davenport, of Paris, France, on "The Significance of the Natural Form and Arrangement of the Dental Arches of Man, with a consideration of the changes which occur as a result of their artificial derangement by Filing, or by the Extraction of Teeth." Accompanying the paper were a great number of large maps and many plaster models, illustrating the ideas advanced by the author.

The paper discussed the benefits derived from a perfect arrangement of the teeth in their occlusion, and the annoyances caused by imperfect articulation. The natural form or arrangement of the teeth was declared best for use and for preservation. The character of the enamel, presenting a hard, smooth surface and completely covering the crowns, was intended for service and protection.

The nearer perfect the articulation, the firmer become the teeth in the jaw. The doctor does not favor extracting as a means for prevention of proximal decay, nor does he consider proximal contact a dangerous condition, or tending to invite caries. He does not find that the loss of a single tooth materially changes the general articulation, unless it happens to be one of the six front teeth. If a lower tooth is removed, the arch will become somewhat flattened and the upper front teeth more prominent, yet the contact will again be complete. Extraction occasions contraction of the dental arches, as shown on maps. Removing the first molar previous to the eruption of the second molar, deprives the mouth of much masticating surface and tends to a tipping forward of the back teeth. Results from extraction of bicuspids, such as irregularity and contraction, were illustrated by diagrams and models. He considers time a good regulator in many cases, and thinks harm is often done by extraction. Before the second molar appears (if removal is necessary) is the safest time to extract the first molar, and is productive of less harm than at other periods. Unsupported teeth are sure to move until support is secured by contact.

Upper molars rotate when moved out of normal position, the posterior cusps inclining outward and forward. Extraction causes contraction, shortens the bite, rotates molars and bicuspids, and lessens masticating surface. There is no sense in extraction to relieve crowded arches with a view of anticipating caries. On the contrary, the teeth can be better kept where contact is general.

The doctor pities those who have lost their first molars, and still more does he pity those who have lost only the two lower first molars, as in the latter case the lower arch becomes contracted and the upper teeth more prominent.

The paper strongly condemns filing between the teeth, which practice it deems pernicious, and gives reasons for so stating. The filed surfaces often are painful, and are more likely to again decay, the decay in such instances extending to and beyond the margin of the gum. They cause annoyance in masticating, are difficult to keep clean, and besides, the spaces thus made close up, leaving them in a worse condition to treat than before. To parties who have suffered the annoyances caused by having their teeth filed apart, artificial teeth may be considered a boon.

Physicians should understand that many physical ailments may be traced to imperfect articulation and imperfect mastication, often the results of injudicious extraction and filing of teeth.

DISCUSSION.

Dr. Geo. S. Allan wished to discuss the paper at length, but was unable to remain later. He took exception to some of the statements of the writer, believing that there are cases where teeth in an overcrowded condition can be better preserved by judicious filing than where coutour fillings are made. He hoped the discussion of the paper would be continued at next meeting.

Dr. F. Y. Clark intimated that the paper and diagrams pictured the results of filing in the worst form. He could refer to many instances where filing or cutting away by the Arthur method, properly done, had been the means of saving many teeth. He liked the paper, although not fully in accord with all its sentiments.

Dr. J. W. Clowes said that the paper was a grand effort to uphold wrong ideas. It had portrayed the worst results, or odds and ends of bungling practice—extreme cases; yet it was evidently intended to convey the idea that such results are the general fruits of good operations as well. This, Dr. Clowes declared, was not the case. He knew from long experience that to make separations properly and intelligently was good practice, and a means of preserving the teeth. His patients were convinced of this fact, and it seems strange that all dentists cannot see it in the same light as himself. Where natural spaces exist, no cavities are to be found between the teeth, but where contact is the rule cavities abound. Separations should be made with judgment, and the double-bevel edge secured, opening freely on palatine or lingual surfaces. Such separations do not favor decay, but on the contrary tend to prevent it, and give opportunities for cleansing. Teeth crowded together leave V-shaped pockets for lodgment of acid-generating secretions, which do the mischief. Preparations of iron and other deleterious agents prescribed by physicians, destroy tooth structure.

Dr. W. H. Dwinelle is disposed to be Eclectic in his practice, but took exceptions to the remarks of the last speaker. To mutilate teeth by filing is to destroy the contour designed by the Creator, the best possible form, and which was intended for service and preservation. He eulogized the paper and complimented the efforts of the author. He objects to extraction except in extreme cases, and favors contour fillings.

At this point it was suggested that, as many guests from other cities were present who probably would not attend the next meeting, the remaining time of the session be occupied exclusively by them, and they were invited to express their views.

Dr. Merriam, of Boston, expressed his admiration of the paper, and fully coincided with the views of the author.

Prof. E. T. Darby, of Philadelphia, thinks there is too much extracting done for purposes of regulating teeth and for preventing decay. He said that a somewhat prominent dentist of Philadelphia has a hobby for removing first permanent molars, doing this on every occasion possible, and apparently on general principles, without taking into consideration circumstances or conditions. Another dentist there runs riot on the subject of removing lateral incisors to relieve crowded arches, and Dr. Darby can readily tell by casual observation when such parties have fallen into this man's hands. He thinks that these two dentists do great harm, and feels that it would be a good thing for Philadelphia if they were—somewhere else ! He presented a plaster model of a mouth, showing ill results from injudicious extraction of the first inferior molars.

Dr. J. T. Codman, of Boston, spoke of the essay as a grand paper, containing a world of truth. He thought it the best paper ever prepared by a dentist, and he was in full accord with all it expressed. The doctor was very emphatic in condemning the practice of separating teeth.

Dr. S. H. Guilford, of Philadelphia, in a very neat address expressed his views of the subject under discussion. He would exercise his best judgment instead of following fixed rules, and be governed by circumstances. He believed that in some cases benefits were to be secured by timely extraction, while sometimes extraction proves an injury. He would carefully consider cases presented for treatment, and do what seemed best, or necessary in each. Dr. Guilford referred to a certain condition of irregularity portrayed on one of the maps, where the upper incisors protruded forward and the cutting edges of the lower incisors were elongated, reaching the gum above where the teeth were closed. He corrected such deformities by introducing a vulcanite plate to cover the roof of the mouth, making it thick in front, or back of the upper incisors. Wearing such an appliance prevents the molars and bicuspids from coming together, thus inducing them to elongate and permitting the upper incisors to be pressed back.

Dr. N. W. Kingsley thought that too much stress was laid on the slight changes of articulation alluded to, or the loss of the little masticating surface occasioned by the removal of a tooth, or even a little tipping of some of the teeth. Many people eat only on one side of their mouth, and do it well; and where one or several teeth are missing they still masticate their food freely and without inconvenience. He would by no means favor extracting teeth indiscriminately, yet cases occurred where extraction was of decided benefit.

President Bogue referred to the labors of Dr. Davenport, the author of the paper, and his efforts to treat the subject fully. He heartily endorsed the views of the writer, especially in regard to the extraction of the first molars and the use of the file. He had seen among the upper classes of Europe many fine sets of teeth in their normal condition, free from defects, that had not been mutilated by dentists.

Dr. J. B. Rich intimated that these might have been exceptional cases. Persons possessing perfect dentures were usually found among the peasants of Europe, or the uncivilized races of different countries.

A vote of thanks was tendered to Dr. Davenport for his very interesting paper, and at a late hour the society adjourned to another apartment for dental exercises of a pleasant character.

